# The comparative efficacy of group- versus home-based exercise programs in patients with ankylosing spondylitis: Protocol for a meta-analysis

**DOI:** 10.1097/MD.0000000000011540

**Published:** 2018-07-20

**Authors:** Hui Liang, Xu Tian, Xiao-Ling Liu, Shu-Ya Wang, Yi Dai, Li Kang, Li-Sai Chen, Li-Fen Jin

**Affiliations:** a Department of Nursing, the First People's Hospital of Yunnan Province; b The Affiliated Hospital of Kunming University of Science and Technology, Kunming, Yunnan, China; c Chongqing Key Laboratory of Translational Research for Cancer Metastasis and Individualized Treatment, Chongqing University Cancer Hospital & Chongqing Cancer Institute & Chongqing Cancer Hospital, Chongqing; d Department of Rheumatology and Immunology, the First People's Hospital of Yunnan Province, Kunming, Yunnan, China.

**Keywords:** ankylosing spondylitis, group-based exercise, home-based exercises, meta-analysis

## Abstract

**Background:**

Ankylosing spondylitis (AS) is an important factor to not only cause employment obstacle, but also result in serious social economic load. Several randomized controlled trials investigated the efficacy of group- versus home-based exercise programs in patients with AS. This systematic review will collect and summarize the available evidence to realize the effectiveness of group- and home-based programs in patients with AS.

**Methods:**

A search in PubMed, Web of Science, EMBASE, and the Cochrane Library will be electronically performed by 2 independent investigators to capture all potential studies comparing group- and home-based in patients with AS. The time limit of search will be from their inception to April 2018. Two independent investigators provide their agreement in presencial meeting for a final selection, and at a later stage, the articles will be reviewed in full-text by the all authors. Quantitative analysis will be performed with Review Manager (RevMan) software (version 5.3.0).

**Results:**

This meta-analysis will provide a high-quality synthesis of current evidence of group- versus home-based exercise programs in patients with AS.

**Conclusion:**

The conclusion of our meta-analysis will provide the evidence which program is an effective intervention for patient with AS.

## 1 Introduction

Ankylosing spondylitis (AS) is a chronic and progressive rheumatic disease, which will make patients to suffer from pain, fatigue, joint destruction, deformity, even disability, loss of joint function, and so on.^[1]^ Issued data illustrated that the overall prevalence is ranging from 0.1% to 1.4% for AS.^[2]^ To date, the etiology of AS is still not fully known, however, published evidences suggested that several aspects such as heredity, drugs and accident may be the contributor to this condition. With the progression of the AS, the patients often suffered from limited motion and deformity of the spine, dysfunction, and poor quality of life (QoL).^[3]^ At the same time, published evidences also reported that patients with inflammatory arthritis in primary care also affected by increased cardiovascular morbidity and mortality.^[4]^ Therefore, AS has been regarded as an important factor to not only cause employment obstacle, but also result in serious social economic burden.^[5]^ And thus, AS patients will also be instructed to receive treatment to reduce pain and morning stiffness, and then to further prevent deformity and maintain correct posture, to also keep physical condition and psychosocial health as well.

At the present, various therapeutic modalities (pharmacological and nonpharmacological) can be used to relieve uncomfortable symptoms and put off disease progression for AS. Despite several promising options has been developed in pharmacological therapy, the lasted recommendations released by the Assessments of SpondyloArthritis International Society and the European League Against Rheumatism (ASAS/EULAR) emphasize that combination of pharmacological including nonsteroidal antiinflammatory drugs (NSAIDs), disease-modified antirheumatic drugs (DMARDs) and tumor necrosis factor (TNF) inhibitor and nonpharmacological treatments including exercise should be the optimal management for AS.^[6,7]^ It is noted that exercise, which is one of the nonpharmacological treatments, positively interferes in all kinds of physical and psychological sides. Several studies suggested that even low intensity physical exercise also obtained considerable reductions in mortality and improved health outcomes among patients when compared with subjects receiving controls.^[8,9]^ For patients with AS, 2 exercise programs including home-based and group-based exercise has been applied in practice, however published evidences found that home-based exercise can effectively improve the health-related QoL and reduce fatigue in patients with AS. More importantly, home-based exercises program is cheaper, easier and more efficient, and thus it has been advised to be as for the promising nonpharmacological option for the treatment of AS.^[10–12]^ It is noted that, however, several studies have been recently performed to examine the effect of group-based exercise program, and found beneficial effects on pain, mobility, function, and disease activity in patients with AS.^[13,14]^ It is difficult to make decision in clinical practice due to home-based exercise program and group-based exercise program all obtained promising results in AS patients.^[15]^ Therefore, we designed the present meta-analysis to investigate whether group-based exercise program is superior to home-based exercise program in terms of disease activity and functional status in patients with AS. We designed this systematic review and meta-analysis on April 6, 2018 and we expected to complete this study by August 30, 2018.

## 2 Methods and design

We designed this protocol for a systematic review and meta-analysis according to the framework constructed in preferred reporting items for systematic reviews and meta-analysis protocols (PRISMA-P) 2015: elaboration and explanation.^[16]^ The systematic review and meta-analysis has been registered in the International Prospective Register of Systematic Reviews (PROSPERO) platform with the number of CRD42018097046. We will perform all statistical analyses in accordance with the recommendations proposed by Cochrane Collaboration (CC).^[17]^

### 2.1 Eligibility criteria

Papers, which population inclusion criteria gathered adult patients with AS diagnosed by a rheumatologist will be chosen. Randomized controlled trials (RCTs) or clinical trials, in which at least one of the groups received the group-based exercises or home-based exercises, will be included. The outcome measures of interest were the Bath Ankylosing Spondylitis Metrology Index (BASMI), the Bath Ankylosing Spondylitis Functional Index (BASFI), the Bath Ankylosing Spondylitis Disease Activity Index (BASDAI), and depression. Participants aged less than 18 years old or with juvenile-onset of AS will be excluded. Review articles, observational studies without controls, case reports, cross-sectional studies, self-controlled studies, and commentaries will also be excluded.

### 2.2 Search strategy

The aim of this meta-analysis is to investigate the comparative effects of home-based exercise related to group-based exercise programs in AS patients. The research team will search 4 electronic databases including PubMed, Web of Science, EMBASE, and the Cochrane Library using combinations of the terms home-based exercise, supervis∗ exercise, group-based exercise, and ankylosing spondylitis. Search terms will be adapted for use with each database. Common keywords and medical subject headings (MeSH) consisted of 2 components: the condition (AS), the intervention (home-based exercise, group exercise or supervis∗ exercise). The time limit of search will be designed from their inception to April 2018. In the present meta-analysis, we will only consider the study published in English language. Moreover, we will check the bibliographies of eligible studies and topic-related review to capture any potential study. If disagreement on citation search was identified, a third senior investigator will be consulted to solve it.

### 2.3 Study selection

Two authors independently will be assigned to screen titles and abstracts in order to identify those studies that meet the inclusion criteria. And then, we will retrieve the full-text of potential studies, and 2 independent investigators will check the eligibility of each study. Any divergency about study selection will be addressed through consulting a third senior investigator.

### 2.4 Data extraction

All captured citations will be imported into EndNote literature management software V.X7. We will then assign 2 reviewers to abstract the basic information and data for the specific outcomes from the eligible studies, such as first author, publication year, age of participants, sample size, details of interventions, and outcomes of interest using this standard data extraction form. We will contact the corresponding author if sufficient data of an eligible study cannot be abstracted from the full text.

### 2.5 Quality assessment

We will assign 2 independent reviewers to appraise the risk of bias from seven domains, including randomization sequence generation, allocation concealment, blinding of participants, blinding of study personnel, blinding of outcome assessors, incomplete outcome data, selective reporting and other bias with the Cochrane risk of bias assessment tool.^[17,18]^ A study will be assigned a risk level of “high risk of bias,” “unclear risk of bias,” or “low risk of bias” according to the match level between the actual information and the evaluation criteria.^[17]^

### 2.6 Statistical analysis

Outcome measures were compared between participants treated with group-based or supervised exercises group and home-based group with each study. The homogeneity among trials was evaluated using *P*-value, and we used the fixed-effects model if there was no evidence of heterogeneity (*I*^2^ < 50%), otherwise a random-effects model was used. Pooled differences in means were calculated and a 2-tailed *P*-value < .05 was considered to indicate statistical significance. All analyses were performed using Review Manger (RevMan) statistical software, version 5.3.0 (Cochrane Collaboration, Copenhagen, Denmark).

### 2.7 Publication bias

For single outcome, we will draw the funnel plot to identify publication bias if the number of studies analyzed is more than 10.^[19]^ Moreover, we will also perform the Egger linear regression test to quantitatively detect the symmetric or a symmetric of funnel plot.^[20]^

## 3 Discussion

AS is a debilitating condition for patients because it will cause several morbid consequences such as joint destruction, deformity, and loss of joint function.^[1]^ Because the etiology of AS has not yet been fully clarified, and thus treatment was performed to only reduce symptoms. Despite several pharmacological options have been developed, some adverse events limit the use of pharmacological therapy. Considered these limits of pharmacological therapy, the ASAS/EULAR recommends combination of pharmacological and non-pharmacological treatments to be the optimal management for AS.^[6,7]^ To date, 2 exercise programs including home-based and group-based exercise can be selected to treat AS patients, however the comparative efficacy of these 2 interventions is not known. Just because of this, it is difficult to make decision in clinical practice due to home-based exercise program and group-based exercise program all obtained promising results in AS patients.^[15]^ Therefore, it is essential to investigate the comparative of group-based exercise program compared to home-based exercise program in patients with AS.

This systematic review and meta-analysis will be one of the first to investigate the comparative efficacy and safety of home-based exercise and group-based exercises programs for patients with AS. The results of the systematic review and meta-analysis will influence evidence based decision making for management of AS patients as it will be fundamental in providing reliable recommendations for AS patients management.

## Author contributions

Hui Liang, Xu Tian, Xiao-Ling Liu, and Li-Fen Jin conceived the study, developed the study criteria. Shu-Ya Wang and Yi Dai searched the literature. Li Kang and Li-Sai Chen analyzed the data. Hui Liang, Xu Tian, Xiao-Ling Liu, and Li-Fen Jin wrote the protocol. Hui Liang and Xu Tian conducted the preliminary search. Xu Tian and Xiao-Ling Liu extracted data. Hui Liang and Li-Fen Jin revised the manuscript. All authors have read, and approved the final manuscript.

**Conceptualization:** Hui Liang, Xu Tian, Xiao-Ling Liu, Li-Fen Jin.

**Data curation:** Xiao-Ling Liu, Shu-Ya Wang, Yi Dai.

**Formal analysis:** Xu Tian, Li-Sai Chen.

**Investigation:** Li Kang, Li-Fen Jin.

**Methodology:** Xu Tian, Li Kang, Li-Sai Chen, Li-Fen Jin.

**Resources:** Xiao-Ling Liu, Shu-Ya Wang, Yi Dai.

**Supervision:** Hui Liang, Xu Tian, Li-Fen Jin.

**Writing – original draft:** Hui Liang, Xu Tian, Xiao-Ling Liu, Li-Fen Jin.

**Writing – review & editing:** Hui Liang, Xu Tian, Li-Fen Jin.
